# Molecular and epidemiological characterization of recurrent *Mycobacterium ulcerans* infections in Benin

**DOI:** 10.1371/journal.pntd.0010053

**Published:** 2021-12-28

**Authors:** Ronald Gnimavo, Alban Besnard, Horace Degnonvi, Juliana Pipoli Da Fonseca, Marie Kempf, Christian Roch Johnson, Alexandra Boccarossa, Yao Télesphore Brou, Laurent Marsollier, Estelle Marion

**Affiliations:** 1 Centre de Diagnostic et de Traitement de l’ulcère de Buruli, Fondation Raoul Follereau, Pobè, Bénin; 2 Univ Angers, Inserm, INCIT, Angers, France; 3 Centre Inter Facultaire de Formation et de Recherche en Environnement pour le Développement Durable (CIFRED), Université d’Abomey Calavi (UAC), Cotonou, Benin; 4 Institut Curie Genomics of Excellence (ICGex) Platform, Institut Curie, Paris, France; 5 Univ Angers, Inserm, CHU Angers, INCIT, Angers, France; 6 CNRS, UMR ESO, Université d’Angers, Angers, France; 7 UMR 228 ESPACE-DEV (IRD, UAG, UM, UR), Station SEAS-OI, Saint Pierre, Ile de la Réunion, France; University of Melbourne, AUSTRALIA

## Abstract

**Background:**

Buruli ulcer is a neglected tropical disease caused by *Mycobacterium ulcerans*, an environmental mycobacterium. Although transmission of *M*. *ulcerans* remains poorly understood, the main identified risk factor for acquiring Buruli ulcer is living in proximity of potentially contaminated water sources. Knowledge about the clinical features of Buruli ulcer and its physiopathology is increasing, but little is known about recurrence due to reinfection.

**Methodology/Principal findings:**

We describe two patients with Buruli ulcer recurrence due to reinfection with *M*. *ulcerans*, as demonstrated by comparisons of DNA from the strains isolated at the time of the first diagnosis and at recurrence. Based on the spatial distribution of *M*. *ulcerans* genotypes in this region and a detailed study of the behavior of these two patients with respect to sources of water as well as water bodies and streams, we formulated hypotheses concerning the sites at which they may have been contaminated.

**Conclusions/Significance:**

Second episodes of Buruli ulcer may occur through reinfection, relapse or a paradoxical reaction. We formally demonstrated that the recurrence in these two patients was due to reinfection. Based on the sites at which the patients reported engaging in activities relating to water, we were able to identify possible sites of contamination. Our findings indicate that the non-random distribution of *M*. *ulcerans* genotypes in this region may provide useful information about activities at risk.

## Author summary

Buruli ulcer is a neglected tropical disease caused by *Mycobacterium ulcerans*, an environmental mycobacterium. Although transmission of *M*. *ulcerans* remains poorly understood, the main identified risk factor for acquiring Buruli ulcer is living in proximity of potentially contaminated water sources. For this infectious disease occurring mainly in Africa, little is known about recurrence due to reinfection. We describe two patients with Buruli ulcer recurrence due to reinfection with *M*. *ulcerans*, as demonstrated by comparisons of DNA from the *M*. *ulcerans* strains isolated at the time of the first and second diagnostics. We formally demonstrated that the recurrence in these two patients was due to reinfection. Based on the sites at which the patients reported engaging in activities relating to unprotected water, we were able to identify possible sites of contamination. Our findings indicate that the non-random distribution of *M*. *ulcerans* genotypes in this region may provide useful information about human activities at risk for contamination.

## Introduction

Buruli ulcer is a neglected tropical disease caused by *Mycobacterium ulcerans*, an environmental mycobacterium. This cutaneous infectious disease occurs principally in the countries of West and Central Africa and in Australia [1]. Although transmission of *M*. *ulcerans* remains poorly understood, the main identified risk factor for acquiring Buruli ulcer is living in proximity of potentially contaminated water sources. One of the probable reservoirs of *M*. *ulcerans* is linked to the aquatic environment and humans are generally contaminated through activities involving sources of water as well as water bodies and streams [1–6]. *M*. *ulcerans* produces a toxin, mycolactone, which mediates host colonization and tissue damage [7–10].

Buruli ulcer is a necrotizing hypodermatitis that begins with a closed lesion (nodule, plaque or edema) forming several weeks or months after the inoculation of the skin with the bacillus. The lesion progresses towards an open, ulcerative lesion that may expand massively, leading to major irreversible functional sequelae [11,12]. Spontaneous healing may occur several months after the onset of infection, but this process may also lead to severe sequelae. In some rural areas of Africa in which the disease is endemic, diagnosis often occurs late. As a result, patients presenting severe forms of the disease still constitute the majority of cases treated. Buruli ulcer is treated by the daily administration of rifampicin and clarithromycin for 56 days, with adaptive wound care and surgery, if required [13,14]. The WHO recommends administering treatment only after biological confirmation of Buruli ulcer has been obtained by PCR, but access to this tool remains difficult in rural endemic areas.

Knowledge of the clinical features of Buruli ulcer and its physiopathology is increasing, but we still know little about recurrence due to reinfection, partly due to the difficulty gaining access to laboratories performing quantitative PCR (qPCR) for confirmation and isolating *M*. *ulcerans* strains by culture. Reinfection, as opposed to relapse or a paradoxical reaction (exacerbated immune reactions directed against dead bacilli), has been reported in only one Buruli ulcer patient to date [15]. We describe here two patients presenting recurrence due to reinfection with *M*. *ulcerans*, as demonstrated by comparing DNA from the strains isolated at the time of the first diagnosis and at recurrence. The spatial distribution of *M*. *ulcerans* genotypes in this region suggested possible hypothesis concerning the sites at which the patients may have been contaminated.

## Materials and methods

### Ethics statement

The Ethics Committee from Benin, named “Comité National d’Ethique pour la Recherche en Santé” gave a favorable opinion, under the number “N°03, November 2019”, to the research project, including child and adult participants. Data collection (including patient clinical data) was also approved by the institutional review board of the CDTLUB and the national Beninese Buruli ulcer control authorities (IRB00006860). A formal written consent was obtained for adult participant or from one of the parents for child participant.

### Data collection

This study was conducted at the *Centre de Diagnostic et de Traitement de la Lèpre et de l’Ulcère de Buruli* (CDTLUB; the Leprosy and Buruli Ulcer Diagnosis and Treatment Center) in Pobè, Benin, which has one of the largest databases of patients with PCR-confirmed Buruli ulcer [11]. Between 2009 and 2018, 1,186 PCR-positive Buruli ulcer patients were diagnosed and treated at this center. Biological confirmation was performed at the molecular laboratory of the CDTLUB Pobè, on swabs or fine-needle aspiration (FNA) samples. Quantitative PCR (qPCR) targeting *M*. *ulcerans* DNA was routinely performed, and a culture was set up to isolate *M*. *ulcerans* strains [16]. Briefly, swabs were rehydrated in 2ml of sterile water. To isolate *M*. *ulcerans* strains, 400μl of swab specimens were decontaminated by the Kubica method, whereas FNA specimens were not because the sampling procedure is considered as sterile. After inoculation onto Lowenstein-Jensen medium, growth was monitored weekly for 5 months. For qPCR analyses, DNA was extracted from 400μl of specimen by NaOH/heat method and purified on QIAquick purification kit. We interviewed two patients with recurrent Buruli ulcer at their homes, and recorded the GPS coordinates of their main water sources and agricultural fields in their presence.

### DNA capture

One sample sequenced in this study corresponded to the DNA extracted from a swab. No isolate was obtained. To sequence this DNA sample, we applied a method of DNA capture before performing a whole genone sequencing. A custom targeted sequence capture array for M. ulcerans was generated using the Roche NimbleGen SeqCap method (Madison, USA), biotinylated DNA probes were designed *in silico* to cover 100% of the genome M. ulcerans reference (GenBank: GCA_000013925.2, 1 chromosome and 1 plasmid). The probes that hybridized to human genome HG38 were removed. Libraries were prepared according to the SeqCap EZ Hyper Cap Workflow by Roche. In resume, 100ng gDNA were fragmented using a Covaris E200 targeting 180-220bp fragment size. We then proceed to prepare libraries using the Kapa Hyper Prep kit as recommended by Roche. We did hybridization of the samples for 64h. After incubation, samples were captured as recommended by the SeqCap EZ Hyper Cap Workflow user guide. The captured DNA was sequenced using a Nextseq 500 Mid Output format, paired-end 150bp.

### Sequencing and NGS analysis

DNA was extracted from pure cultures, and libraries were prepared and sequenced, as previously described [17]. Details of the clinical isolates are shown in S1 Table. Reads from the DNA capture data mapping with the GRCh38 human genome were removed using Bowtie 2.2.7 [18]. We used snippy 4.6.0 [19] to detect SNPs and we kept only SNPs with sufficient coverage (10) on all our 177 strains. We used Phyml 3.3 [20] and R 4.1.1 [21] with the ggtree package [22] to visualize the phylogenetic tree, and a map was generated with an OpenStreetMap layer. The model used in the analysis is the HKY85 model. We submitted generated reads to the National Center for Biotechnology Information Sequence (Bioproject ID PRJNA743744) and previous genome data used in this study are available under the Bioproject ID PRJNA499075. The isolate from the patient 2- first episode (DNA sequence number: 1193–13), was already sequenced in the Bioprojet ID PRJNA499075, while the three other isolates (1173–15, 150-5-09 and 226–18) were sequenced in this study.

## Results

During the 2009–2018 period, two patients presented a recurrence of Buruli ulcer, with an interval of about five years between lesions. Below, we provide: (i) a clinical presentation of the two patients, (ii) the results of *M*. *ulcerans* sequencing and spatial analysis, and (iii) an analysis of human water-related activities.

### Clinical presentation

The clinical characteristics of the patients are presented in Table 1.

**Table 1:** Description of the two episodes of Buruli ulcer in the two patients.

	Patient 1		Patient 2	
	Male, 8 years old at the time of the first episode		Female, 35 years old at the time of the first episode	
	1^st^ episode: 2009	2^nd^ episode: 2015	1^st^ episode: 2014	2^nd^ episode: 2018
**Location**	Behind the knee	Right forearm	Right leg	Left forearm
**Type of lesion**	Cat 1	Cat 1	Cat 3	Cat 1
**Description**	Plaque (2.5 x 3 cm) with ulcer (0.5 cm)	Nodule and plaque (4.5 x 5 cm)	Plaque (16 x 12 cm) with ulcer (6 cm)	Ulcer (4 x 3 cm)
**Laboratory confirmation**	PCR^+^ / ZN^+^	PCR^+^	PCR^+^	PCR^+^ / ZN^+^
**Date of consultation**	01/22/2009	08/05/2015	12/21/2013	02/19/2018
**Antibiotic treatment**	R + C 56 days	R + S 56 days	R + S 56 days	R + C 56 days
**Hospitalization (days)**	56	56	150	0
**Type of sample**	Swab	FNA	swab	swab
**Origin of DNA**	DNA extracted from swab	culture	culture	culture
**DNA sequence number**	150-5-09	1173–15	1793–13	226–18
**Genotype**	8	5	4	7
**SNP differences**		37		40

FNA = Fine needle aspiration

ZN = Ziehl-Neelsen staining

C = clarithromycin S = streptomycin and R = rifampicin

#### Patient 1

In 2009, an eight-year-old boy was treated for a Buruli ulcer lesion of less than 5 cm in diameter located behind the knee (Patient 1, Episode 1: P1E1). The small ulcer was surrounded by a plaque. Swabs taken under the undermined edge were positive for *M*. *ulcerans* DNA. No microorganism grew in the cultures set up after sample decontamination. The patient was treated daily with rifampicin and clarithromycin, for 56 days. Surgery was not necessary and the patient was discharged from hospital with a completely healed lesion at the end of the antibiotic treatment. In 2015, five years and seven months after the first diagnosis, the child presented a small plaque (<5 cm) on the right forearm (P1E2). FNA was performed on the center of the lesion. The aspiration fluid tested positive for *M*. *ulcerans* DNA by PCR. An isolate of *M*. *ulcerans* was also obtained from this lesion. The patient was treated daily with streptomycin and rifampicin for 56 days. At the end of treatment, he was discharged from the hospital with a small weeping lesion.

#### Patient 2

A 35-year-old woman consulted for the first time in 2014, for a large lesion located on the right leg. The lesion was a plaque (16 x 12 cm) with ulceration (6 x 6 cm) (P2E1). A second site was also identified, in the form of a non-ulcerative plaque with a diameter of 6 cm on the right thigh. A swab was taken from the ulcerative lesion and a fine-needle aspiration was performed on the non-ulcerative lesion. Both samples were positive for *M*. *ulcerans* DNA and a culture was obtained from the swab sample. The patient was treated daily with rifampicin and streptomycin for 56 days. Surgery was required for debridement and to prepare the wound for a skin graft to facilitate healing. On day 150 after the initiation of antibiotic treatment, the lesions were considered to be completely healed. In 2018, five years and two months after the first diagnosis, the woman consulted for the onset of a small lesion on the left forearm, with a small ulcer (4 x 3cm) and edema (P2E2). PCR was positive for *M*. *ulcerans* DNA and an isolate of *M*. *ulcerans* was obtained from the sample. The patient was treated daily with rifampicin and clarithromycin, without the need for hospitalization. At the end of antibiotic treatment, the lesion was considered to be completely healed, and there was no need for surgery.

### Analysis of *M*. *ulcerans* genome sequencing

**Fig 1:**
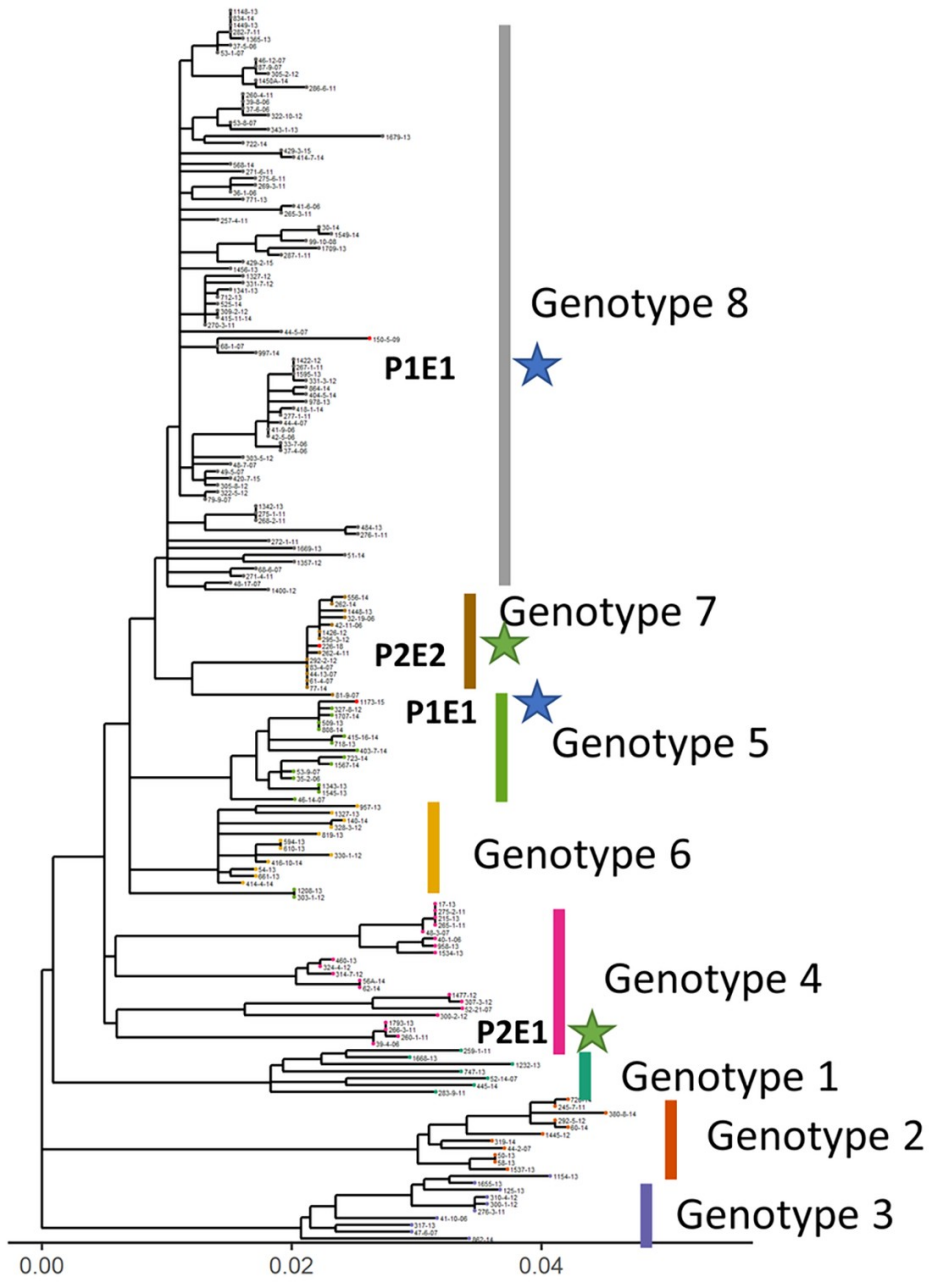
Positions of the strains responsible for the first and second episodes of Buruli ulcer in the two patients on a phylogenetic tree of *M*. *ulcerans*. The tree contains 175 *M*. *ulcerans* strains from the eight genotypes identified in this endemic area. The strains of patient 1 (P1E1 first episode and P1E2 second episode) and patient 2 (P2E1 first episode and P2E2 second episode) are indicated with blue and green stars, respectively. The tree was rooted with the Agy99 strain (not shown for clarity) and used a total of 986 SNP.

We compared the *M*. *ulcerans* genome sequences obtained between the first and second Buruli ulcer episodes, for both patients. Based on our previous study analyzing 179 *M*. *ulcerans* sequences from strains isolated from the same region in Benin, in which we identified eight distinct genotypes [17], we analyzed the SNPs of the four *M*. *ulcerans* strains and constructed a phylogenetic tree, to position them amongst the other sequenced strains (Fig 1). Among the 996 SNPs used in the analysis, the PE1E1 was 37 SNPs separated from P1E2 and the P2E1 was 40 SNPs separated from P2E2. For patient 1, the strain that caused the first Buruli ulcer belonged to genotype 8, whereas the second strain belonged to genotype 5. For patient 2, the first strain belonged to genotype 4, and the second to genotype 7. These analyses clearly demonstrated that the second episodes in these patients were caused by a different *M*. *ulcerans* strain, distinguishable from that responsible for the first episode.

**Fig 2:**
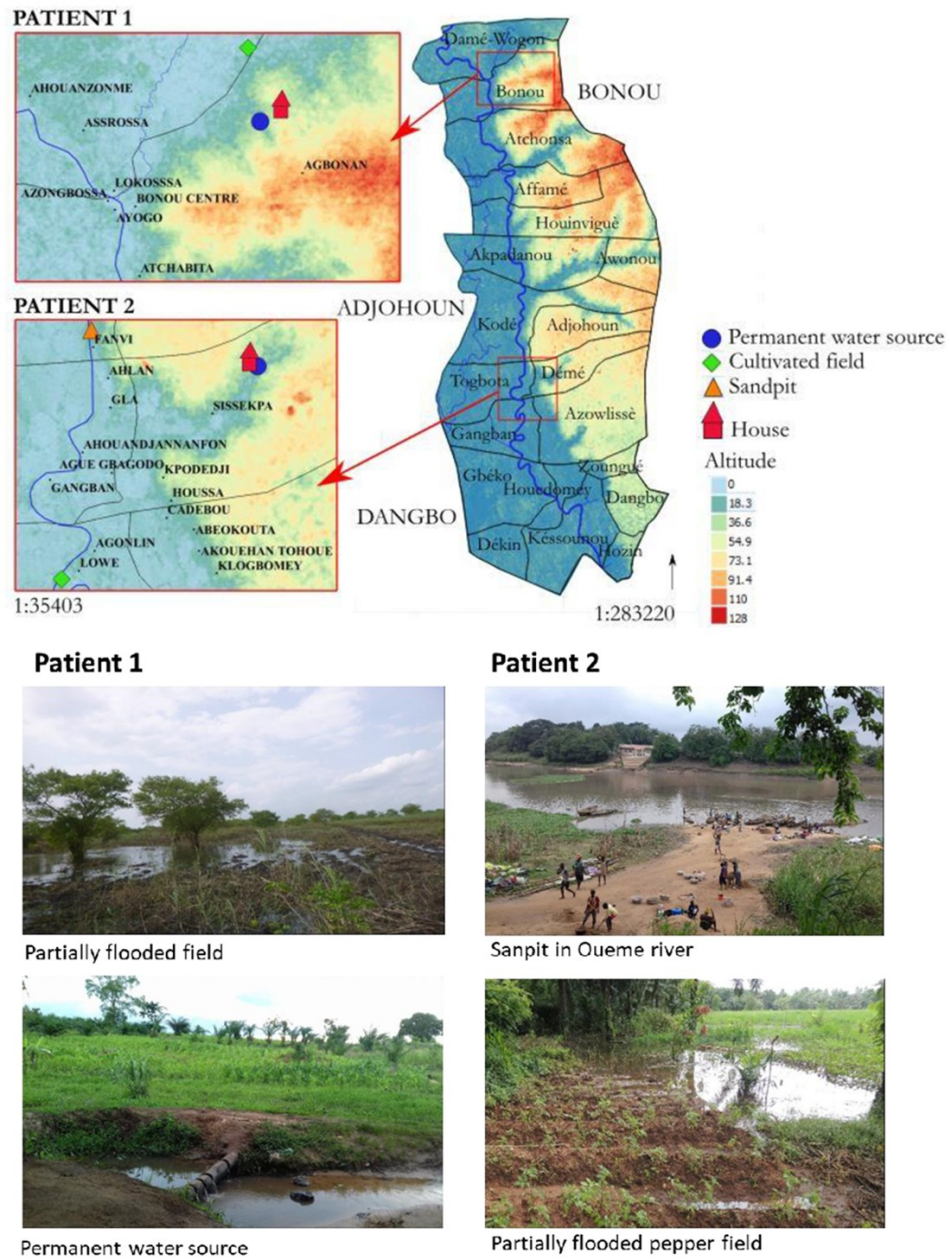
Geolocalization and images of patients’ homes, workplaces and permanent water sources in Ouémé, Benin. Datas for the altitude and land surface are available on https://earthexplorer.usgs.gov/ (NASA) https://apps.sentinel-hub.com/eo-browser/?zoom=7&lat=8.21149&lng=0.27466&themeId=DEFAULT-THEME&visualizationUrl=https%3A%2F%2Fservices.sentinel-hub.com%2Fogc%2Fwms%2Ff2068f4f-3c75-42cf-84a1-42948340a846&datasetId=S1_AWS_IW_VVVH&fromTime=2021-12-15T00%3A00%3A00.000Z&toTime=2021-12-15T23%3A59%3A59.999Z&layerId=IW-DV-VV-DECIBEL-GAMMA0-ORTHORECTIFIED, and https://apps.sentinel-hub.com/eo-browser/ (European Spatial Agency) https://apps.sentinel-hub.com/eo-browser/?zoom=7&lat=8.21149&lng=0.27466&themeId=DEFAULT-THEME&visualizationUrl=https%3A%2F%2Fservices.sentinel-hub.com%2Fogc%2Fwms%2F6448ffd0-56c5-4601-bed7-242bf75d97db&datasetId=DEM_COPERNICUS_30&fromTime=2021-12-16T00%3A00%3A00.000Z&toTime=2021-12-16T23%3A59%3A59.999Z&layerId=GRAYSCALE. Datas for the administrative boundary were provided by Ministry of Territory Planning of Benin https://data.humdata.org/dataset/benin-administrative-boundaries#.

We recently showed that *M*. *ulcerans* genotypes were not randomly distributed along the Ouéme river [17]. The two strains obtained from patient 1 belonged to genotypes found mostly in Northern Ouéme, where the patient lived with his parents. The first strain obtained from patient 2 belonged to a genotype present in Northern Ouéme and the second strain belonged to a genotype found almost exclusively in Southern Ouéme. Patient 2 lived in-between these two areas (Fig 2). We interviews both patients, asking them about their principal activities involving water, as a means of identifying the potential activities at risk for these two patients.

### Analysis of patient behavior

#### Patient 1

During and between the first and second Buruli ulcer episodes, the boy lived with his parents, in the same house, and went to the same school. The house was located in Agbonan village, Monsekpota locality, in Northern Ouémé (Fig 2). The water used by the family (essentially for drinking, washing, bathing) came from either a water drilling or from a permanent water source. The boy did not travel to other places or countries between the two Buruli ulcer episodes. He helped his family in the fields (mainly cassava and corn production). The fields are located within 1.5 km of the family’s home and are flooded for part of the year. The family cultivates the fields when the water recedes, but water persists in some areas. The patient had no direct contact with the Ouéme River, which is located much farther from the family’s home (3.5 km). The permanent water source used by the family is not stagnant (Fig 2). The place of highest risk for this patient would therefore appear to be the cultivated fields, in which there is some stagnant water and waterholes.

#### Patient 2

The woman lived with her husband and children in the village of Sissekpa in Adjohoun municipality (Fig 2). She remained in this area, without traveling outside the county) between the two Buruli ulcer episodes. The water she used (for drinking, washing, bathing, and cooking) came from a permanent water source. During the first episode, the woman was working at a sandpit close to the Ouéme river, and with her husband in the family’s oil palm fields, which are not subject to flooding at any time in the year (Fig 2). After the first Buruli ulcer episode, she stopped working at the sandpit and found employment as an agricultural worker in Southern Ouéme (Dannou village, Gangban municipality). She was involved in cultivating pepper in the fields of the farm, which are flooded for part of the year and require irrigation at other times during the year (Fig 2). She watered the crop with water from a waterhole in the middle of the field that remained after the flood waters subsided. The contamination of this patient with two different strains—one from the northern part of Ouéme and the other from the southern part of this *county*—can be explained by the change in her working activities related to water, with a move from the north to the south of Ouéme between the two Buruli ulcer episodes.

## Discussion

Next-generation sequencing (NGS) is a powerful technology that is increasingly being used in microbiology to improve our understandings between bacterial and their hosts. This technology has already been used to analyze reinfections in patients infected with monomorphic bacteria, such as *Mycobacterium tuberculosis* or *Clostridium difficile*, and to distinguish between reinfections and relapses. In both the cases reported here, in the face of a probable recurrence of Buruli ulcer due to re-infection, with no evidence of relapse, the clinicians decided to treat the second infection with the reference antibiotic treatment for Buruli ulcer [13,14]. Second episodes of Buruli ulcer may be caused by reinfection, but they may also be due to relapse or a paradoxical reaction [15,23,24]. However, as compliance with antibiotic treatment was closely monitored, at the hospital, for the first episodes of both patients, the risk of relapse was very low. Paradoxical reactions are a differential diagnosis for lesions of this type, particularly if positive results are obtained in PCR tests for *M*. *ulcerans* [24]. Only a positive culture from this lesion could rule out the diagnostic of a paradoxical reaction. However, *M*. *ulcerans* grows very slowly (two to six months) and the positive culture rate is low. The clinicians could not, therefore, wait for the results of such analyses before deciding on the best course of patient management. We were able to rule out paradoxical reactions retrospectively, however, because *M*. *ulcerans* strains were isolated by culture during the second episode.

We demonstrated, in this study, the possibility of sequencing *M*. *ulcerans* directly from a clinical sample without the need to obtain a clinical isolate. The method is based on the specific capture allowing the concentration of *M*. *ulcerans* DNA while reducing the contaminating DNA. It is therefore possible to consider the use of this method for tracking *M*. *ulcerans* from environmental sources, as already done for Borrelia [25].

*M*. *ulcerans* whole-genome sequencing demonstrated the occurrence of reinfection in both cases, thereby confirming the clinical diagnosis. Indeed, for both patients, the genotypes of the strains responsible for the first and second episodes were different. The number of SNPs separated the two episodes is 37 for patient 1 and 40 for patient 2 respectively. The difference is similar to the first re-infection described in a closed endemic area in Benin where the SNP difference between the 2 strains was 20 [15]. Furthermore, we recently showed that *M*. *ulcerans* strains were not randomly distributed other this endemic area. Based on the sites at which the patients engaged in water-related activities, it was possible to identify likely sites of contamination through such activities. Our results indicate that the non-random distribution of *M*. *ulcerans* genotypes can be used as a tool for investigating activities at risk.

The description of these two cases of reinfection highlights the lack of immune protection afforded by a first episode of Buruli ulcer. However, based on findings for other mycobacterial diseases (tuberculosis, leprosy), it is not surprising that primary infection does not enhance immune protection against a second exposure [26,27]. The only vaccine currently available for mycobacterial disease is BCG, which does not provide full protection against *M*. *tuberculosis* [28]. Furthermore, studies in a mouse model of *M*. *ulcerans* infection showed that mice displaying spontaneous healing after a first infection were not protected against a second challenge with *M*. *ulcerans* [29]. Efforts to develop a vaccine for this neglected tropical disease must consider all these elements.

It is possible that these two patients present a genetic susceptibility to *M*. *ulcerans* infection, but their blood tests revealed no abnormalities or suspicion of immunodepression. A role for host genetics in susceptibility to Buruli ulcer has long been suggested and a recent study identified lncRNAs and the autophagy pathway as critical factors in the development of Buruli ulcer [30,31]. Further whole-genome sequencing analyses should be performed on these two patients, to investigate this possibility further.

## Supporting information

S1 Table Lists of all *M*. *ulcerans* genomes used in the study, including the SSR accession number. (XLSX)
